# Utility of immature platelet fraction in the Sysmex XN‐1000V for the differential diagnosis of central and peripheral thrombocytopenia in dogs and cats

**DOI:** 10.1111/jvim.17074

**Published:** 2024-04-15

**Authors:** Alejandro Perez‐Ecija, Carmen Martinez, Julio Fernandez‐Castañer, Francisco J. Mendoza

**Affiliations:** ^1^ Department of Animal Medicine and Surgery University of Cordoba, Campus Rabanales Cordoba Spain; ^2^ Veterinary Teaching Hospital University of Cordoba, Campus Rabanales Cordoba Spain

**Keywords:** hematology, hemostasis, platelets, thrombopoiesis

## Abstract

**Background:**

The immature platelet fraction (IPF), a parameter obtained by the Sysmex XN‐1000V analyzer, is used in humans to differentiate between central (CEN) and peripheral (PER) thrombocytopenia (TP) but has not been evaluated in small animals.

**Objectives:**

Compare IPF between healthy, clinical non‐TP and TP dogs and cats, study IPF in different causes of TP in dogs and cats and, establish IPF reference intervals (RIs), and study the effect of age and sex on IPF in healthy dogs and cats.

**Animals:**

A total of 3281 dogs and 726 cats.

**Methods:**

Retrospective review of medical records. Animals were classified as nonthrombocytopenic (healthy group and group of clinical patients without TP [NTP]) or TP. These latter animals were subclassified as pseudothrombocytopenia (PSE), CEN and PER, based on evaluation of platelet clumps, estimated platelet count in blood smears and final diagnosis. Blood samples were evaluated using a Sysmex XN‐1000V with a specific platelet channel (PLT‐F).

**Results:**

The IPF was significantly different between each subtype of TP in both species. Immature platelet fractions <6.9% in dogs or 13.6% in cats, once PSE has been eliminated by review of blood smears, are indicative of CEN. Reference intervals for IPF were 0.5%‐8% in healthy dogs and 1%‐40.3% in healthy cats.

**Conclusions and Clinical Importance:**

We determined that IPF can differentiate between CEN and PER in dogs and cats, guiding additional testing and avoiding more invasive procedures (bone marrow sampling). A blood smear always should be evaluated to rule out platelet clumping.

**Abbreviations**
ANOVA: analysis of variance
ASVCP: American Society for Veterinary Clinical Pathology
AUC: area under the curve
CEN: central thrombocytopenia
CD‐61: cluster of differentiation 61
CI: confidence interval
EDTA: ethylenediaminetetraacetic acid
IPF: immature platelet fraction
IPFc: immature platelet count
IQR: interquartile range
MPV: mean platelet volume
NTP: nonthrombocytopenic clinical patients
PCR: polymerase chain reaction
PCT: plateletcrit
PDW: platelet distribution width
PER: peripheral thrombocytopenia
P‐LCR: platelet‐large cell ratio
PLT: platelet count
PLT‐F: fluorescence platelet channel
PSE: pseudothrombocytopenia
RI: reference interval
RNA: ribonucleic acid
ROC: receiver operating characteristic
TP: thrombocytopenia

## 1 INTRODUCTION

Thrombocytopenia (TP) is a common laboratory abnormality in small animals.^1^ Thrombocytopenic patients can be prone to life‐threatening hemorrhage or spontaneous bleeding during surgical procedures.^2^ The differential diagnosis of thrombocytopenia is a complex process that requires a combination of patient history, clinical signs, hematologic testing including measurement of specific platelet parameters, and ancillary diagnostic tests such as clotting times, as well as serological, biomolecular, and imaging procedures.

Platelet clumps caused by improper collection or mixing of the sample and EDTA‐dependent pseudothrombocytopenia (PSE) also can cause a decreased platelet count (PLT) in healthy animals.^3^ This artifact can be identified as the cause of thrombocytopenia by evaluating blood smears and identifying the presence of platelet aggregates.

Once TP is confirmed, it can be classified either as central (CEN), caused by bone marrow failure to produce sufficient platelets (eg, neoplasia, infectious agents, hormones, toxins, radiation, drugs), or peripheral (PER), caused by increased consumption, destruction or sequestration of platelets (eg, immune‐mediated TP, splenopathy), based on the presence of active thrombopoiesis.^3^ Thus, assessment of megakaryocyte numbers in a bone marrow aspirate or biopsy traditionally has been required to differentiate between these causes.^2^ However, bone marrow sampling is an expensive, time‐consuming, and invasive procedure, subject to variable interpretation depending on the pathologist's experience and sample quality.^4^

The Sysmex XN‐1000V analyzer has a specific optic‐fluorescent channel for platelets (PLT‐F) which uses a specific fluorochrome and uses a higher sample volume. In humans, the use of this channel improves the accuracy of counts in case of interference and at lower PLT ranges.^5^ Additionally, an oxazine‐based fluorescent dye recognizes RNA content in the platelets, and those with increased side fluorescent signal are counted as immature platelet fraction (IPF), previously known as reticulated platelets.^6^ These RNA‐rich platelets have been released recently into circulation and potentially are analogous to reticulocytes reflecting erythropoiesis.

Immature platelet fraction is used in human medicine as a useful diagnostic parameter to differentiate between CEN and PER.^7^, ^8^ This parameter correlates with megakaryocytic activity and platelets recognized as immature by flow cytometry (using double staining with thiazole orange for RNA and cluster of differentiation 61 antigen [CD61]).^8^, ^9^ Although measurement of IPF using the XN‐1000V recently has been validated in veterinary medicine,^10^ information about its utility in the differential diagnosis of thrombocytopenia in dogs and cats is lacking.

Therefore our objectives were to: compare IPF using the XN‐1000V analyzer among healthy, clinical patients without TP and clinical patients with TP, determine the utility of IPF in the XN‐1000V in the differential diagnosis between CEN and PER in dogs and cats, characterize the IPF reference intervals (RIs) using the XN‐1000V, and evaluate the effect of age and sex on IPF in healthy dogs and cats.

## 2 MATERIALS AND METHODS

### 2.1 Case selection and classification

Animals were retrospectively selected from those referred to the Veterinary Teaching Hospital of the University of Cordoba between January 2021 and May 2023 with complete medical records and a CBC using the XN‐1000V analyzer (Sysmex Corporation, Kobe, Japan).

**FIGURE 1:**
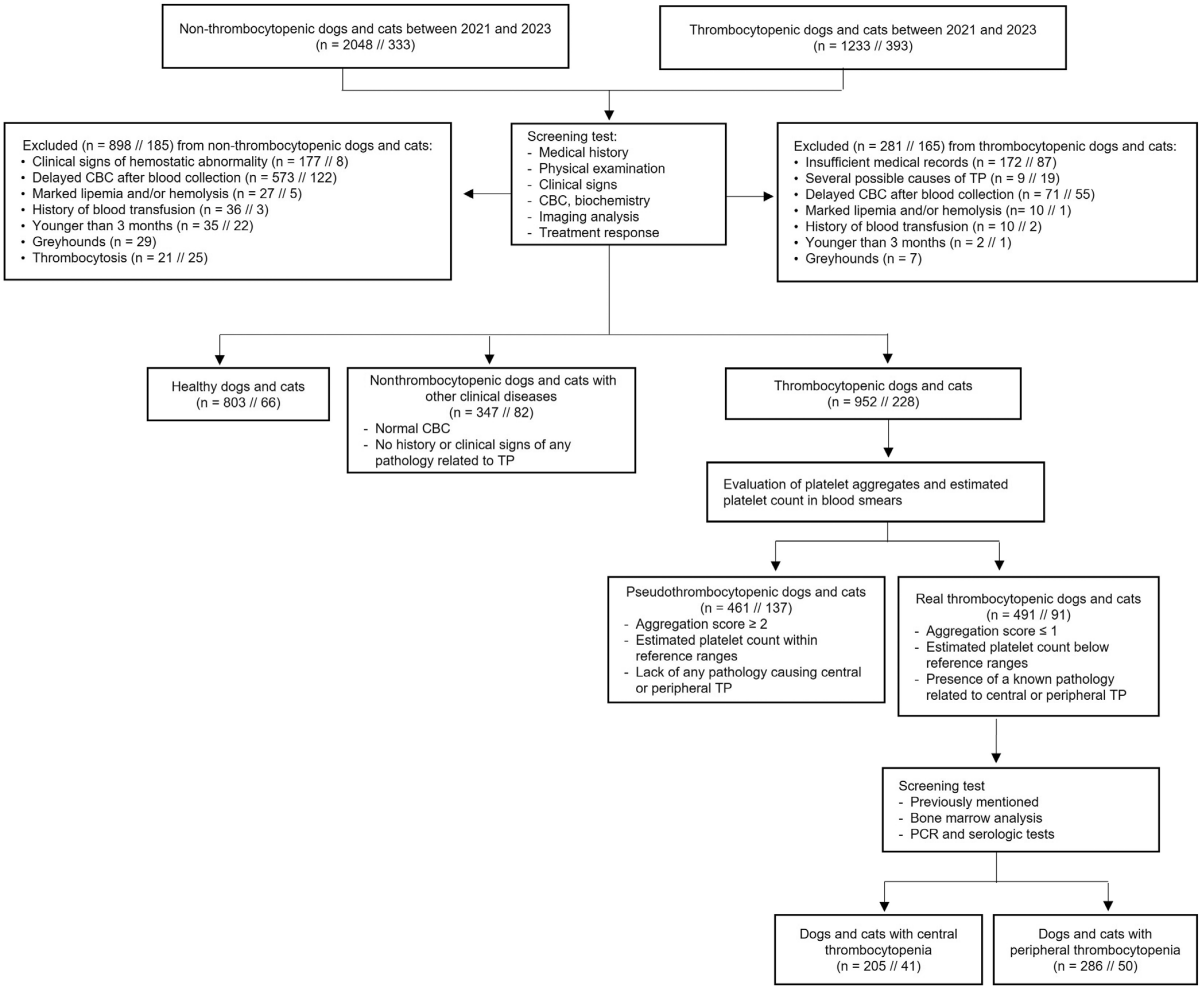
Flow diagram of case enrollment in the study.

Dogs and cats were divided (Figure 1) into healthy (samples from annual health assessments of healthy patients), clinical patients without thrombocytopenia (NTP) and TP groups. Animals were considered healthy based on normal clinical history and physical examination, and hematology results within the RI at our institution: red blood cell count, 5‐8.6 × 10^6^/μL; hematocrit, 35%‐52%; hemoglobin, 12‐20 g/dL; platelet count, 200‐500 × 10^3^/μL in dogs and red blood cell count, 5‐12 × 10^6^/μL; hematocrit, 30%‐45%; hemoglobin, 10‐15 g/dL; platelet count, 250‐550 × 10^3^/μL in cats.

Animals diagnosed with disorders not related to platelets and with CBC results within reference ranges were included in the NTP group (Figure 1).

Thrombocytopenic animals were selected from those with platelet counts below RI (Figure 1). A peripheral blood smear was used to estimate platelet count and evaluate platelet aggregation. Briefly, the average number of platelets in 10 oil immersion fields was multiplied by 15 × 10^3^ to calculate the estimated platelet count.^3^ Platelet aggregation was graded using a semiquantitative scale (0 = no aggregates to 4+ = maximal aggregation) as previously described.^11^ Cases with a score ≥2, an estimated platelet count within RIs, and no suspicion of any disease causing platelet disorders were classified as PSE. Animals with automatic and estimated platelet counts below RIs, a platelet aggregation score ≤1 and a diagnosis related to known causes of CEN or PER were considered as truly thrombocytopenic. These latter animals were subclassified (Figure 1) depending on history, clinical signs, results from complementary diagnostic tests (serum biochemistry, bone marrow examination, blood PCR for infectious causes, thoracic radiographs, abdominal ultrasonography), and treatment response into the PER or CEN groups.

Briefly, patients without any sign of bone marrow compromise and with a final diagnosis of any of the following diseases: disseminated intravascular coagulation (prolonged prothrombin and partial activated thromboplastin times with increased D‐dimer concentration in the absence of other cause of TP), rickettsial infections (based on positive PCR or serological test in absence of other diagnosis), hypersplenism or splenomegaly (based on ultrasonography in absence of other diagnosis) or immune‐mediated thrombocytopenia (absence of any other diagnosis leading to PER and response to immunosuppressive treatment) were included in the PER group. On the other hand, animals without any disease proven to cause PER and either bone marrow cytology or histology results suggesting bone marrow failure or concurrent nonregenerative anemia and leukopenia (eg, toxic bone marrow suppression, myelophthisis, myelofibrosis) were included in the CEN group. Concerning bone marrow evaluation, <5 megakaryocytes per particle (evaluating 20 × 10 objective fields) was considered a sign of central failure by cytology, whereas <2 megakaryocytes per intertrabecular space (evaluating the same fields) was used in bone marrow histology, according to previously published guidelines.^12^, ^13^ Every smear and slide was evaluated by a diplomate pathologist (A.P).

Because of economic restraints and owner preferences, not all of the listed diagnostic tests were performed in every case.

Exclusion criteria were the following (Figure 1): delayed CBC analysis after blood collection, lack of definitive diagnosis, history of blood transfusion within 30 days before sampling, animals <3 months old (to avoid unrecognized hematological abnormalities associated with age‐related changes),^14^ and Greyhounds (because of specific breed‐related platelet ranges).^3^ No repeated analyses from the same patient were included and only the initial CBC was considered. Only cases with sufficient clinical information and diagnostic results to allow a definitive classification of the type of thrombocytopenia (CEN vs PER) were included in the study.

The Institutional Animal Care and Use Committee of the Veterinary Teaching Hospital of the University of Cordoba approved this study and owner consent was obtained.

### 2.2 Sampling and measurements

Blood samples were collected by cephalic or jugular venipuncture into an EDTA‐containing tube and analyzed within 30 minutes using a Sysmex XN‐1000V analyzer (Sysmex Corporation, Kobe, Japan). The instrument's graphical information was inspected and the following parameters were retrieved: platelet counts using the optic channel (PLT), mean platelet volume (MPV), plateletcrit (PCT), platelet distribution width (PDW), platelet‐large cell ratio (P‐LCR), immature platelet fraction (IPF) and immature platelet count (IPFc).

Samples with lipemia or hemolysis were excluded. Internal quality assessment was performed weekly using 2 levels of the manufacturer's quality control material (XNCHECK Level 1 & Level 2; Sysmex Corporation, Kobe, Japan).

### 2.3 Statistical analysis

Normality was assessed using the Kolmogorov‐Smirnov test. Results were expressed as mean ± SD or median and interquartile range (IQR, 25th‐75th percentiles) as appropriate. The median and percentiles were calculated using Tukey's Hinges test.

Groups were compared using the Kruskal‐Wallis test with Dunn's post hoc test or 1‐way ANOVA with Tukey’ post hoc test depending on normality. Spearman's or Pearson's coefficients were used to determine correlations between parameters as appropriate.

Receiver operating characteristic (ROC) curve analysis was performed to determine the sensitivity, specificity, and cut‐off values of IPF for identifying CEN vs PER. Use of ROC curves to determine the performance of a new test in diseases without a clear gold standard, although widely used, can suffer from bias. Thus, these results should be interpreted with caution.

Following recommendations from the American Society of Veterinary Clinical Pathology, RIs were obtained using a robust or nonparametric method as appropriate and dedicated software (Reference Value Advisor v. 2.1. freeware, available at: http://www.biostat.envt.fr/reference-value-advisor/, accessed July 27, 2023) and are presented as 2‐sided 90% CIs.^15^ Only data from healthy animals were used to calculate the RIs.

For the third objective, animals were grouped based on sex and age (<5 years old; 5‐10 years old; >10 years old).

Statistical analyses were performed using statistical software (GraphPad Prism 9, San Diego, California) and values with *P* < .05 were considered significant.

## 3 RESULTS

### 3.1 IPF and other platelet parameters in healthy, NTP, and TP dogs

**FIGURE 2:**
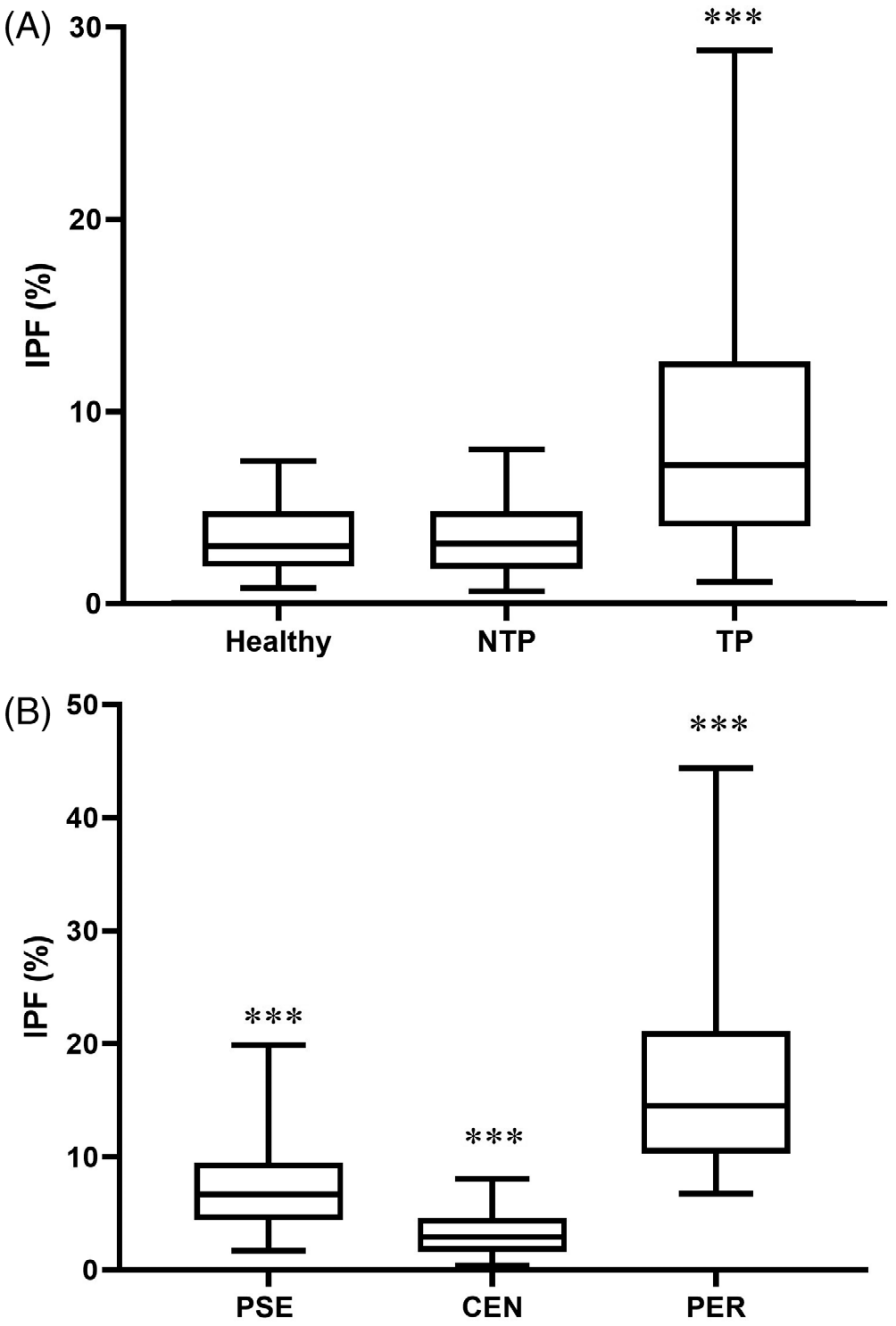
Box and whisker plot of immature platelet fraction (IPF) percentages in dogs grouped by thrombocyte status: (A) healthy (n = 803), clinical patients without thrombocytopenia (NTP, n = 347), and thrombocytopenic dogs (TP, n = 952); (B) pseudothrombocytopenia (PSE, n = 461), central thrombocytopenia (CEN, n = 205), and peripheral thrombocytopenia (PER, n = 286). The box represents the first and third quartiles, the middle line represents the median, and the whiskers represent the 5%‐95% range. *P < .05; **P < .01; ***P < .0001 vs other groups.

A total of 1150 (of 2048) dogs had platelet counts within RIs and fulfilled the inclusion criteria, with 803 animals included in the healthy group and 347 in the NTP group (Figure 1). A total of 952 (of 1233) dogs were included in the TP group. Results for IPF along with other platelet parameters are shown in Table 1 and Figure 2A.

**TABLE 1:** Platelet parameters in dogs (n = 2102).

	Nonthrombocytopenic dogs (n = 1150)		Thrombocytopenic dogs (n = 952)		
	Healthy (n = 803)	NTP (n = 347)	PSE (n = 461)	CEN (n = 205)	PER (n = 286)
PLT (10^3^/μL)	318 (145)	353 (189)	119 (80)*	124 (74)*	128 (70)*
MPV (fL)	10.3 (1.3)	10.4 (1.1)	11.0 ± 1.2*	11.1 ± 1.1*	11.3 ± 1.4*
PDW (fL)	12.7 ± 2.1	12.7 ± 2.4	14.0 (3.0)*	13.0 (3.5)*	14.2 (2.2)*
PCT (%)	0.28 (0.1)	0.33 (0.2)	0.09 (0.1)*	0.09 (0.1)*	0.08 (0.1)*
P‐LCR (%)	29.6 ± 8.2	29.2 ± 8.8	36.9 ± 10.8*	36.7 ± 10.9*	39.6 ± 13.3* ^,a,b^
IPF (%)	3.00 (2.9)	3.10 (3.0)	6.70 (5.1)* ^,b,c^	2.90 (3.0)^a,c^	14.5 (10.8)* ^,a,b^
IPFc (10^3^/μL)	10.1 (8.7)	11.0 (10.4)	7.65 (7.2)* ^,b,c^	3.30 (4.0)* ^,a,c^	15.6 (12.6)* ^,a,b^

*Note*: Data are expressed as median (IQR) or mean ± SD, according to distribution.

Abbreviations: CEN, central thrombocytopenia; IPF, immature platelet fraction; IPFc, immature platelet count; MPV, mean platelet volume; NTP, clinical patients without thrombocytopenia; PCT, plateletcrit; PDW, platelet distribution width; PER, peripheral thrombocytopenia; P‐LCR, platelet‐large cell ratio; PSE, pseudothrombocytopenia.

* *P* < .05 vs healthy and NTP groups. ^a^ *P* < .05 vs PSE. ^b^ *P* < .05 vs CEN. ^c^ *P* < .05 vs PER.

Neither IPF nor IPFc were different (*P* > .24) between healthy and NTP groups. No significant differences were found in PLT, MPV, PDW, PCT or P‐LCR (*P* > .07) between these groups. Immature platelet fraction was significantly higher (*P* < .0001) and IPFc significantly lower (*P* < .0001) in TP dogs compared with healthy and NTP groups. Both PLT and PCT were significantly (*P* < .001) lower, whereas MPV, P‐LCR and PDW were significantly (*P* < .0001) higher in TP compared with the other groups.

The RI for IPF was between 0.5% and 8% in healthy dogs, whereas the range for IPFc was 1.9‐26.8 × 10^3^/μL. Frequency distribution is compiled in Supplementary Figure 1. No significant differences were observed related to sex or age (Supplementary Tables 1 and 2).

### 3.2 IPF and other platelet parameters in different subtypes of TP in dogs

A total of 461 dogs (of 952) were included in the PSE group, whereas 205 were subclassified as CEN and 286 dogs were included in the PER group (of 491; Figure 1).

Both IPF and IPFc were significantly different (*P* < .0001) between all groups (Table 1 and Figure 2B). No significant differences were found for platelet count (*P* > .99), PCT (*P* > .99), PDW (*P* > .21) or MPV (*P* > .89) between TP groups (Table 1). The P‐LCR was significantly higher (*P* < .01) in PER as compared with other groups (Table 1).

No correlations were found between IPF and platelet counts, or any other platelet parameter (Supplementary Table 3).

### 3.3 IPF and other platelet parameters in healthy, NTP, and TP cats

**FIGURE 3:**
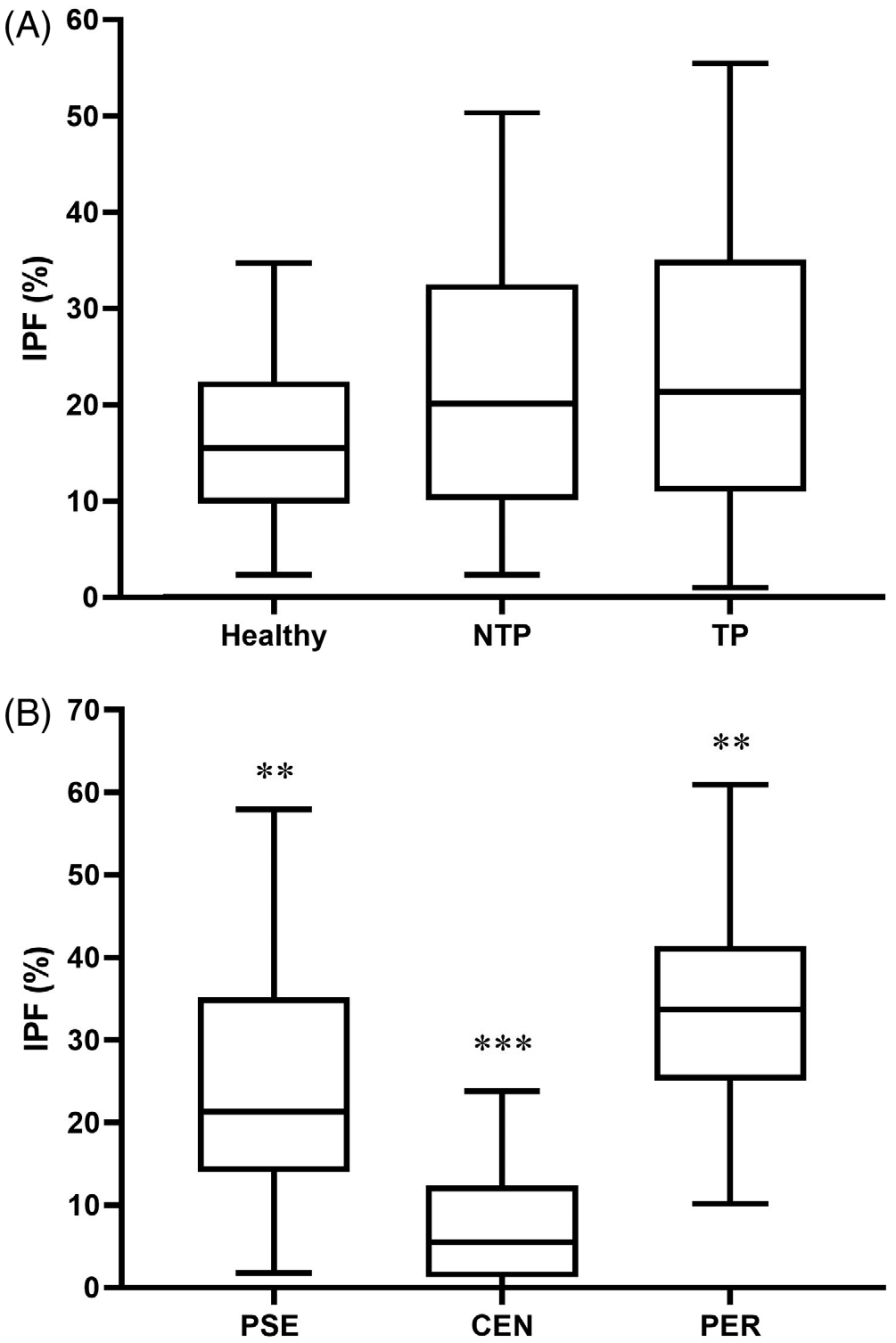
Box and whisker plot of immature platelet fraction (IPF) percentages in cats grouped by thrombocyte status: (A) healthy (n = 66), clinical patients without thrombocytopenia (NTP, n = 82), and thrombocytopenic cats (TP, n = 228); (B) pseudothrombocytopenia (PSE, n = 137), central thrombocytopenia (CEN, n = 41), and peripheral thrombocytopenia (PER, n = 50). The box represents the first and third quartiles, the middle line represents the median, and the whiskers represent the 5%‐95% range. *P < .05; **P < .01; ***P < .0001 vs other groups.

A total of 148 cats (of 333) were included in the nonthrombocytopenic groups, with 66 considered healthy and 82 as NTP. The TP group consisted of 228 cats (of 393). Many samples from thrombocytopenic cats were flagged because of platelet abnormal distribution. Thus, parameters obtained by impedance were considered unreliable and only data from the optic and fluorescence channels were analyzed. Results for IPF, IPFc and platelet counts are shown in Table 2 and Figure 3A.

**TABLE 2:** Platelet parameters in cats (n = 376).

	Nonthrombocytopenic cats (n = 148)		Thrombocytopenic cats (n = 228)		
	Healthy (n = 66)	NTP (n = 82)	PSE (n = 137)	CEN (n = 41)	PER (n = 50)
PLT (10^3^/μL)	347 (111)	407 (333)	139 (108)*	142 (83)*	127 (93)*
IPF (%)	16.4 ± 9.78	20.1 ± 22	21.3 (21.1)^b,c^	5.50 (11.1)* ^,a,c^	33.7 (16.3)* ^,a,b^
IPFc (10^3^/μL)	57.7 (52.2)	83.1 (101)	25.4 (34.5)* ^,b,c^	8.57 (17.2)* ^,a,c^	38.7 (38.6)* ^,a,b^

*Note*: Data are expressed as median (IQR) or mean ± SD, according to distribution.

Abbreviations: CEN, central thrombocytopenia; IPF, immature platelet fraction; IPFc, immature platelet count; NTP, clinical patients without thrombocytopenia; PER, peripheral thrombocytopenia; PSE, pseudothrombocytopenia.

* *P* < .05 vs healthy and NTP groups. ^a^ *P* < .05 vs PSE. ^b^ *P* < .05 vs CEN. ^c^ *P* < .05 vs PER.

No significant differences were found in platelet counts, IPF or IPFc among healthy, NTP and TP cats (*P* > .07).

Reference range for IPF was between 1% and 40.3% in healthy cats, whereas range for IPFc was 4‐156 × 10^3^/μL. Frequency distribution is presented in Supplementary Figure 2. No significant differences were observed related to sex or age group (Supplementary Tables 4 and 5).

### 3.4 IPF and other platelet parameters in different subtypes of TP in cats

A total of 137 cats (of 228) cats were considered PSE, whereas 41 and 50 were included in the CEN and PER groups, respectively (Figure 1).

IPF and IPFc (*P* < .01) were significantly different among TP groups, with CEN having lower percentages and counts (Table 2 and Figure 3B). Platelet counts (*P* > .99) were not significantly different between groups.

No correlations were found between IPF and platelet counts (Supplementary Table 6).

### 3.5 Cut‐off value for the differential diagnosis between CEN and PER in dogs and cats

According to the ROC curve, IPF (%) was accurate for differentiating between CEN and PER, both in dogs and cats (Supplementary Figure 3). The cut‐offs, sensitivity, specificity, and area under the ROC curve of IPF are shown in the Supplementary Table 7. In dogs, P‐LCR lacked sensitivity and specificity for this differential diagnosis and a reliable cut‐off could not be established (Supplementary Figure 3 and Table 7).

## 4 DISCUSSION

We demonstrated that IPF can differentiate between subtypes of TP in dogs and cats. The IPF can be used in dogs and cats to detect active thrombopoiesis and guide further diagnostic tests or procedures (ie, bone marrow sampling can be avoided or prioritized in some cases depending on results). Thus, measurement of IPF by the Sysmex XN‐1000V can be considered a rapid and reliable technique that could be integrated into the diagnostic routine for thrombocytopenic patients.

Our main objective was to evaluate the use of IPF to differentiate subtypes of TP, with special interest in discerning between CEN and PER. The IPF was significantly higher in dogs and cats with PER compared with those affected by bone marrow pathologies. This result reflects a higher platelet production and hence platelet turnover in cases where the main cause of TP involves destruction, consumption or sequestering of platelets or some combination of these, without bone marrow involvement. This finding also has been observed in several studies in human medicine comparing immune‐mediated TP or thrombotic thrombocytopenic purpura to bone marrow defects altering thrombopoiesis.^7^, ^16^, ^17^ In veterinary medicine, a single previous study has shown higher numbers of reticulated platelets in dogs with destruction or consumption pathophysiology, although few animals were included in that study.^4^

Pseudothrombocytopenic animals had higher IPF compared with patients with CEN (although significantly lower compared with PER). The increase of IPF caused by platelet aggregation has been observed previously in both human and veterinary medicine.^10^, ^18^ Because values in animals with PSE overlapped those of other subtypes of TP, this parameter should not be used in isolation, and a blood smear should be evaluated in TP patients. Once PSE was eliminated, IPF could differentiate between CEN and PER with high sensitivity and specificity, using cut‐offs of 6.9% and 13.6% in dogs and cats, respectively. Our cut‐offs in dogs are similar to those used in humans to differentiate between immune‐mediated TP and aplastic thrombocytopenia.^7^, ^16^ The IPF is also useful in human medicine to monitor thrombocytopenic patients during treatment, as a predictive marker of platelet recovery (ie, normalization of IPF during remission in immune‐mediated TP).^17^ Similar findings have been described in a dog using the Sysmex XT2000iV.^19^ Additional studies using a higher number of patients or comparing the response of IPF to different treatments in this disease could be valuable.

Because immature platelets usually are larger than mature platelets, MPV and PDW have been used as surrogate markers for active platelet production.^20^, ^21^ However, these parameters are obtained by impedance, and their validity is negatively affected by low platelet counts as well as schistocytes, microcytes or other particles with a volume similar to platelets.^22^ Moreover, impedance methods are commonly known to be unreliable in samples from cats because of clumping and size overlapping with erythrocytes.^23^ Because we did not find significant differences between subtypes of TP in dogs with regard to MPV or PDW and many samples from cats showed abnormal distributions and flags in the impedance channel (and thus data from this channel were considered unreliable in this species), this approach should be avoided. Although immature platelets usually are larger, IPF is based on fluorescent signals. Thus, there is only a partial correlation with MPV.^24^ The P‐LCR is an impedance parameter that indicates the percentage of circulating large‐sized platelets. In our study, dogs with PER had higher P‐LCR compared with the other groups. Nonetheless, when we tried to obtain a cut‐off to differentiate between CEN and PER in dogs, sensitivity and specificity were markedly low because of overlapping between groups. Thus, this parameter should not be used for this purpose.

Our third objective was to establish RIs for IPF and IPFc in healthy dogs and cats using the Sysmex XN‐1000V analyzer. We separated NTP animals from healthy animals so as to avoid bias. Nonetheless, no significant differences were found either in IPF or in other platelet parameters between the 2 groups, in accordance with a previous study using flow cytometry.^2^ Because IPF is measured using different dyes and techniques in different analyzers, comparisons should be made with caution. Most dogs showed relatively low values and CIs for both upper and lower limits were narrow. Our reference ranges are similar to those reported using the same analyzer in a preliminary study of 44 dogs without PSE.^10^ Moreover, they also are similar to those observed in human medicine,^17^, ^25^ as well as those obtained in dogs using several analyzers with manual gating options or thiazole orange and anti‐CD61 labeling using flow cytometry.^2^, ^26^, ^27^ In our case, using the Sysmex XN‐1000V, it was not necessary to optimize or move any threshold or gating window, which is an important clinical and laboratory convenience. To our knowledge, a single study using 17 healthy cats has reported reticulated platelet data (Jensen AK, Nielsen LN, Bach M, et al. Characterization of reticulated platelets in cats with cardiovascular disease. ECVIM‐CA online Congress, 2021). The IPF values in normal cats were more widespread in our study and also compared with healthy dogs. Although the CIs for the upper limit in cats had high uncertainty, they fulfilled the criteria for being considered informative.^15^ The reason behind this idiosyncrasy should be studied further.

No differences were observed based on sex in IPF, either in healthy cats or in dogs. This finding is similar to what has been observed in human medicine using Sysmex XN series hematology analyzers.^25^ We did not find significant differences in IPF values based on age. In human neonates, specific IPF ranges have been established and variations in this parameter have been observed during maturation.^28^ Because we discarded these patients in our experimental design, it will be interesting to study IPF values in puppies and kittens of different ages. Although we did not evaluate the effect of breed on IPF, no Cavalier King Charles Spaniel was included in our study, and thus the effect of idiopathic asymptomatic thrombocytopenia in this breed should be further investigated.

Our study had some limitations. First, some causes of TP, such as immune‐mediated TP or that caused by *Ehrlichia* spp., can combine both megakaryocyte failure and high platelet destruction.^29^ Because we only included animals with proven bone marrow failure (based on cytology, biopsy or concurrent pancytopenia) in the CEN group, it could be argued that their thrombocytopenia was mostly caused by megakaryocyte damage. Second, we limited our study to differences between CEN and PER, without analyzing in more detail variations in IPF among specific diseases in each subtype of TP (eg, primary vs secondary immune‐mediated TP). Although doing so could have been clinically interesting, we were aware of the difficulties in reliably differentiating or ruling out some of these diseases. Third, many animals were excluded from our study population because of failure of follow‐up or incomplete diagnostic evaluation. Finally, the actual effect of aggregates in the platelet count of pseudothrombocytopenic animals is difficult to calculate with certainty. Although we combined 2 methods in the blood smear and considered the presence or lack of pathology that could cause true TP, we cannot completely rule out that some of these animals could have been misplaced in other groups.

In conclusion, we demonstrated that IPF in the Sysmex XN‐1000V is a useful tool in the diagnosis of TP in dogs and cats, differentiating between CEN and PER. In addition, specific and sensitive cut‐offs for differentiating between both diagnoses were established in both species. Because pseudothrombocytopenic samples have overlapping results with other causes, a blood smear always should be inspected in animals with low platelet counts, and samples with clumping should be discarded before evaluating this parameter. Our newly established RIs for IPF and IPFc were not influenced by sex or age in both species.

## CONFLICT OF INTEREST DECLARATION

Authors declare no conflict of interest.

## OFF‐LABEL ANTIMICROBIAL DECLARATION

Authors declare no off‐label use of antimicrobials.

## INSTITUTIONAL ANIMAL CARE AND USE COMMITTEE (IACUC) OR OTHER APPROVAL DECLARATION

Approved by the Welfare Committee of Animal Experimentation of the Veterinary Teaching Hospital of the University of Cordoba (2021PI/02, approval date: February 8, 2021). Animals were handled according to national guidelines for research animals.

## HUMAN ETHICS APPROVAL DECLARATION

Authors declare human ethics approval was not needed for this study.

## Supporting information


**Supplementary Figure 1.** Frequency distributions and reference intervals for IPF (A) and IPFc (B) in healthy dogs. The observed distribution is represented by the vertical black columns, whereas the red curve is the fitted distribution. Reference limits are drawn as vertical blue lines. Dotted bar surrounding those limits are the 90% confidence intervals.

**Supplementary Figure 2.** Frequency distributions and reference intervals for IPF (A) and IPFc (B) in healthy cats. The observed distribution is represented by the vertical black columns, whereas the red curve is the fitted distribution. Reference limits are drawn as vertical blue lines. Dotted bar surrounding those limits are the 90% confidence intervals.

**Supplementary Figure 3.** Receiver operating characteristic curve of immature platelet fraction (IPF; dogs: continuous line; cats: dashed line) and platelet‐large cell ratio (P‐LCR; dogs: dotted line) for the differential diagnosis between central and peripheral thrombocytopenia.

**Supplementary Table 1.** Immature platelet fraction (IPF) in healthy dogs grouped by sex.

**Supplementary Table 2.** Immature platelet fraction (IPF) in healthy dogs grouped by age.

**Supplementary Table 3.** Correlations between platelet parameters in healthy (A) and thrombocytopenic (B) dogs.

**Supplementary Table 4.** Immature platelet fraction (IPF) in healthy cats grouped by sex.

**Supplementary Table 5.** Immature platelet fraction (IPF) in healthy cats grouped by age.

**Supplementary Table 6.** Correlations between platelet parameters in healthy (A) and thrombocytopenic (B) cats.

**Supplementary Table 7.** Cut‐off values of platelet parameters by receiver operating characteristic (ROC) curve analysis for the diagnosis of central thrombocytopenia in dogs and cats.
