# Supplementary figures and images for: Utility of immature platelet fraction in the Sysmex XN‐1000V for the differential diagnosis of central and peripheral thrombocytopenia in dogs and cats
Source: J Vet Intern Med. 2024 Apr 15;38(3):1512–9. doi: 10.1111/jvim.17074 (PMC11099766; doi:10.1111/jvim.17074)

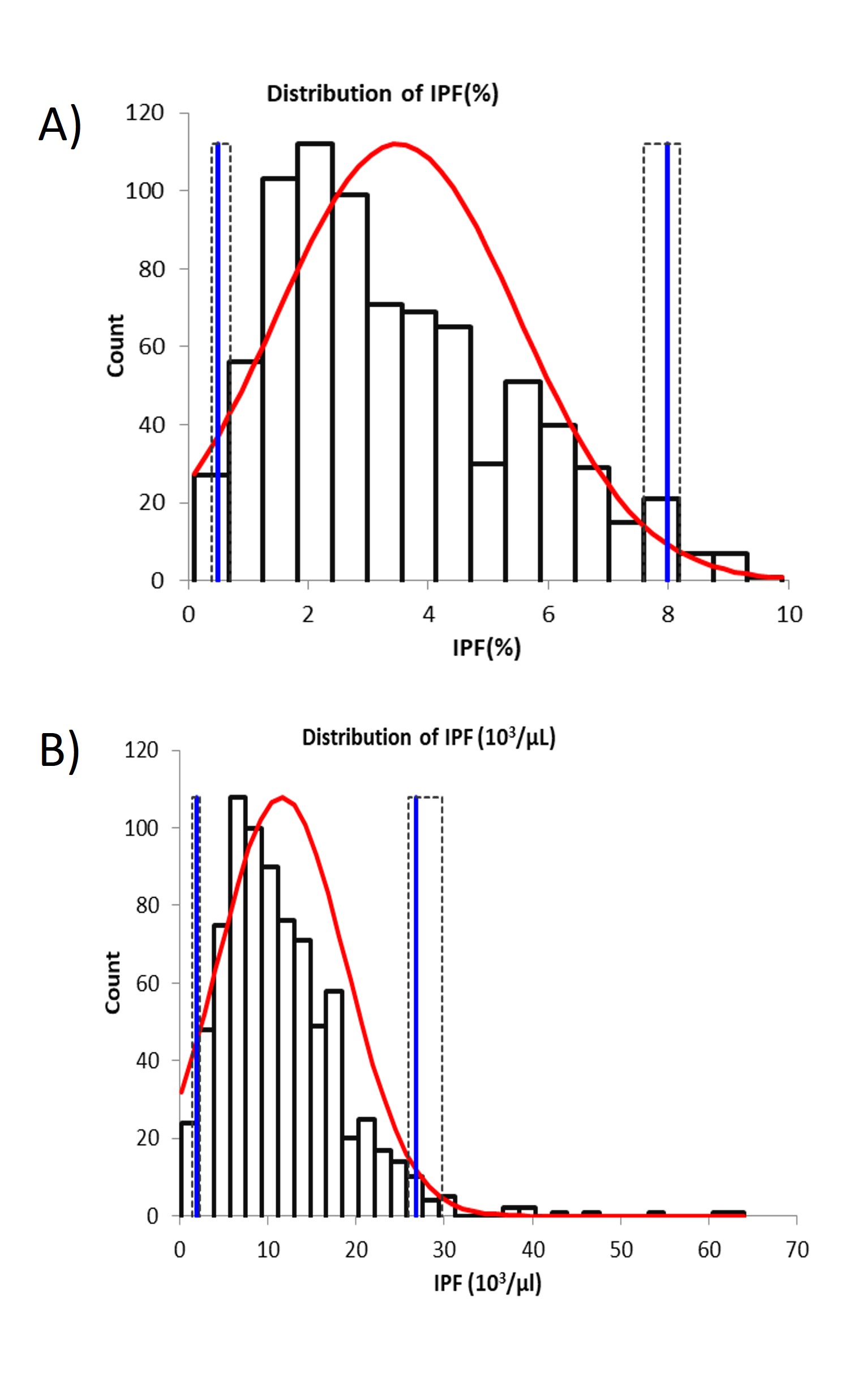

Supplement: Supplementary file 1 — Supplementary Figure 1. Frequency distributions and reference intervals for IPF (A) and IPFc (B) in healthy dogs. The observed distribution is represented by the vertical black columns, whereas the red curve is the fitted distribution. Reference limits are drawn as vertical blue lines. Dotted bar surrounding those limits are the 90% confidence intervals. [file JVIM-38-1512-s010.jpg]

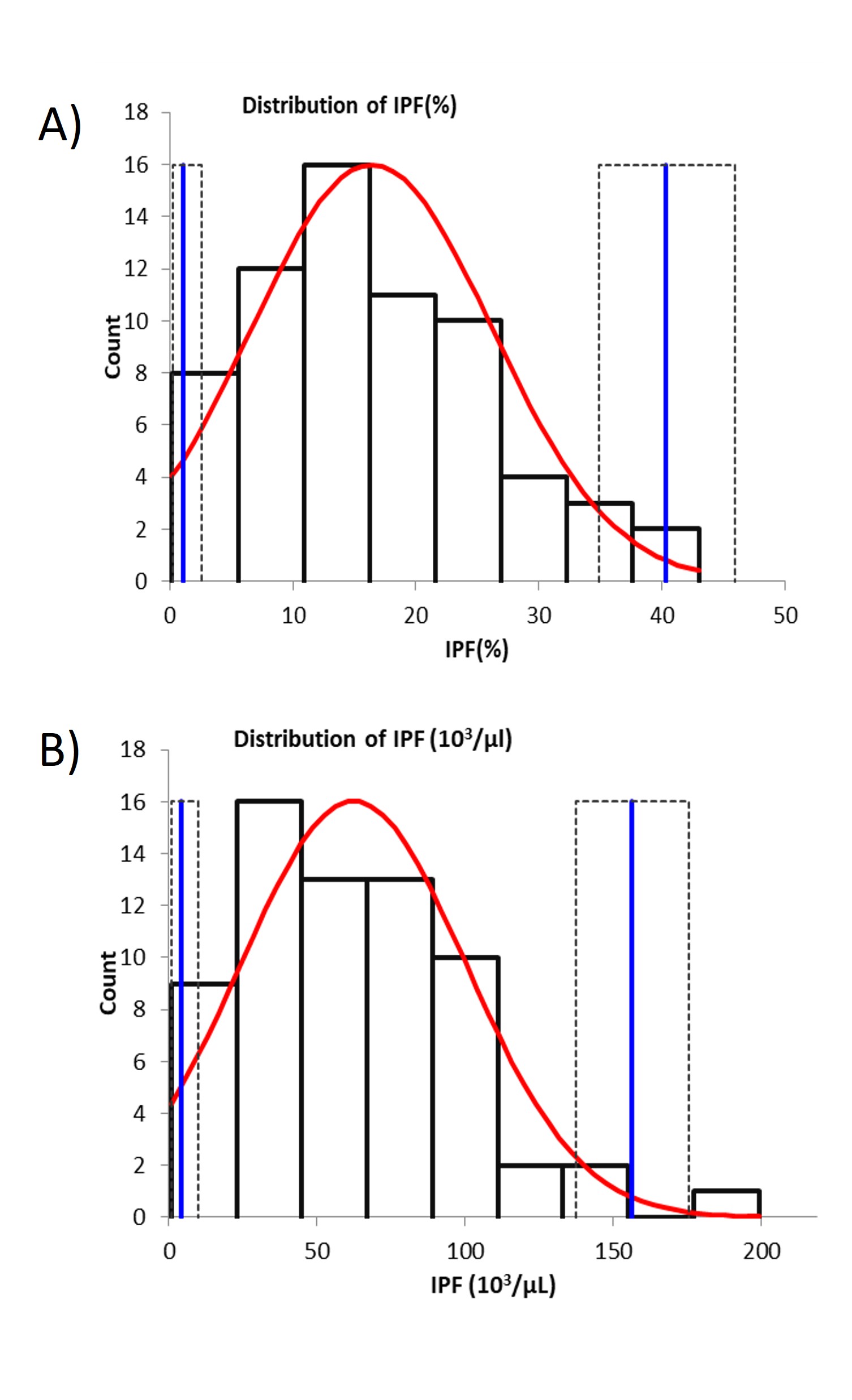

Supplement: Supplementary file 2 — Supplementary Figure 2. Frequency distributions and reference intervals for IPF (A) and IPFc (B) in healthy cats. The observed distribution is represented by the vertical black columns, whereas the red curve is the fitted distribution. Reference limits are drawn as vertical blue lines. Dotted bar surrounding those limits are the 90% confidence intervals. [file JVIM-38-1512-s005.jpg]

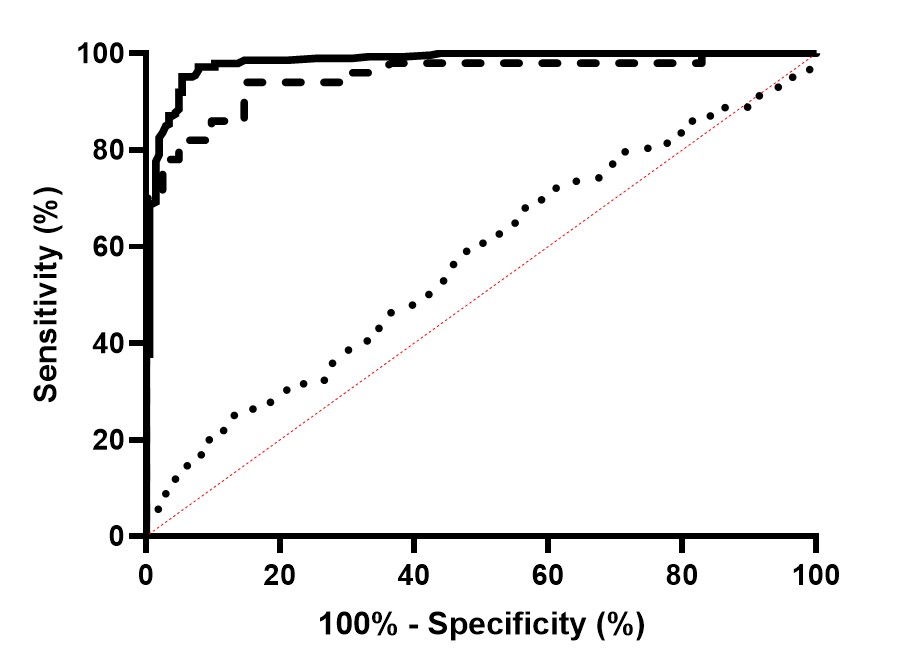

Supplement: Supplementary file 3 — Supplementary Figure 3. Receiver operating characteristic curve of immature platelet fraction (IPF; dogs: continuous line; cats: dashed line) and platelet‐large cell ratio (P‐LCR; dogs: dotted line) for the differential diagnosis between central and peripheral thrombocytopenia. [file JVIM-38-1512-s009.jpg]
