# Supplementary material for: Utility of immature platelet fraction in the Sysmex XN‐1000V for the differential diagnosis of central and peripheral thrombocytopenia in dogs and cats
Source: J Vet Intern Med. 2024 Apr 15;38(3):1512–9. doi: 10.1111/jvim.17074 (PMC11099766; doi:10.1111/jvim.17074)
Supplement: Supplementary file 4 — Supplementary Table 1. Immature platelet fraction (IPF) in healthy dogs grouped by sex. [file JVIM-38-1512-s006.docx]

**Supplementary Table 1.** **Immature platelet fraction in healthy dogs grouped by sex.**

|  | **Healthy dogs** | |
| --- | --- | --- |
|  | **Male**  **(n=317)** | **Female**  **(n=486)** |
| IPF (%) | 3.0 (2.8) | 3.0 (3.1) |
| IPFc (10^3^/µL) | 9.9 (9.2) | 10.2 (8.5) |

Data are expressed as median (interquartile range). IPF, immature platelet fraction; IPFc, immature platelet count. ^*^P < .05 vs the other sex.
