# Supplementary material for: Utility of immature platelet fraction in the Sysmex XN‐1000V for the differential diagnosis of central and peripheral thrombocytopenia in dogs and cats
Source: J Vet Intern Med. 2024 Apr 15;38(3):1512–9. doi: 10.1111/jvim.17074 (PMC11099766; doi:10.1111/jvim.17074)
Supplement: Supplementary file 5 — Supplementary Table 2. Immature platelet fraction (IPF) in healthy dogs grouped by age. [file JVIM-38-1512-s007.docx]

**Supplementary Table 2.** **Immature platelet fraction in healthy dogs grouped by age.**

|  | **Healthy dogs** | | |
| --- | --- | --- | --- |
|  | **Group 1:**  **<5 yo**  **(n=252)** | **Group 2:**  **5-10 yo**  **(n=303)** | **Group 3:**  **>10 yo**  **(n=248)** |
| IPF (%) | 2.9 (2.8) | 2.9 (2.6) | 3.2 (3.0) |
| IPFc (10^3^/µL) | 10.0 (8.3) | 9.8 (9.0) | 10.7 (8.6) |

Data are expressed as median (interquartile range). IPF, immature platelet fraction; IPFc, immature platelet count. ^*^P < .05 vs other groups.
