# Supplementary material for: Utility of immature platelet fraction in the Sysmex XN‐1000V for the differential diagnosis of central and peripheral thrombocytopenia in dogs and cats
Source: J Vet Intern Med. 2024 Apr 15;38(3):1512–9. doi: 10.1111/jvim.17074 (PMC11099766; doi:10.1111/jvim.17074)
Supplement: Supplementary file 6 — Supplementary Table 3. Correlations between platelet parameters in healthy (A) and thrombocytopenic (B) dogs. [file JVIM-38-1512-s001.docx]

**Supplementary Table 3.** **Correlations between platelet parameters in healthy (A) and thrombocytopenic (B) dogs.**

A)

|  | PLT (10^3^/µL) | IPF (%) | IPFc (10^3^/µL) | PDW (fL) | MPV (fL) | P-LCR (%) | PCT (%) |
| --- | --- | --- | --- | --- | --- | --- | --- |
| PLT (10^3^/µL) | 1.00 | - 0.22 | 0.28 | - 0.24 | - 0.23 | - 0.24 | 0.75 |
| IPF (%) |  | 1.00 | 0.85 | 0.48 | 0.49 | 0.50 | - 0.17 |
| IPFc (10^3^/µL) |  |  | 1.00 | 0.32 | 0.36 | 0.36 | 0.20 |
| PDW (fL) |  |  |  | 1.00 | 0.93 | 0.95 | - 0.14 |
| MPV (fL) |  |  |  |  | 1.00 | 0.99 | - 0.09 |
| P-LCR (%) |  |  |  |  |  | 1.00 | - 0.12 |

Data are expressed as Spearman’ or Pearson’ correlation coefficient, based on distribution. IPF, immature platelet fraction; IPFc, immature platelet count; MPV, mean platelet volume; PDW, platelet distribution width; PCT, plateletcrit; P-LCR, platelet-large cell ratio.

B)

|  | PLT (10^3^/µL) | IPF (%) | IPFc (10^3^/µL) | PDW (fL) | MPV (fL) | P-LCR (%) | PCT (%) |
| --- | --- | --- | --- | --- | --- | --- | --- |
| PLT (10^3^/µL) | 1.00 | - 0.14 | 0.41 | 0.04 | 0.10 | 0.02 | 0.83 |
| IPF (%) |  | 1.00 | 0.79 | 0.37 | 0.33 | 0.36 | - 0.17 |
| IPFc (10^3^/µL) |  |  | 1.00 | 0.37 | 0.38 | 0.37 | 0.31 |
| PDW (fL) |  |  |  | 1.00 | 0.84 | 0.87 | 0.10 |
| MPV (fL) |  |  |  |  | 1.00 | 0.98 | 0.22 |
| P-LCR (%) |  |  |  |  |  | 1.00 | 0.12 |

Data are expressed as Spearman’ or Pearson’ correlation coefficient, based on distribution. IPF, immature platelet fraction; IPFc, immature platelet count; MPV, mean platelet volume; PDW, platelet distribution width; PCT, plateletcrit; P-LCR, platelet-large cell ratio.
