# Supplementary material for: Utility of immature platelet fraction in the Sysmex XN‐1000V for the differential diagnosis of central and peripheral thrombocytopenia in dogs and cats
Source: J Vet Intern Med. 2024 Apr 15;38(3):1512–9. doi: 10.1111/jvim.17074 (PMC11099766; doi:10.1111/jvim.17074)
Supplement: Supplementary file 7 — Supplementary Table 4. Immature platelet fraction (IPF) in healthy cats grouped by sex. [file JVIM-38-1512-s004.docx]

**Supplementary Table 4.** **Immature platelet fraction in healthy cats grouped by sex.**

|  | **Healthy cats** | |
| --- | --- | --- |
|  | **Male**  **(n=19)** | **Female**  **(n=47)** |
| IPF (%) | 18.5 ± 10 | 15.5 ± 9.4 |
| IPFc (10^3^/µL) | 66.1 ± 34 | 60.7 ± 39 |

Data are expressed as mean and SD. IPF, immature platelet fraction; IPFc, immature platelet count. ^*^P < .05 vs the other sex.
