# Supplementary material for: Utility of immature platelet fraction in the Sysmex XN‐1000V for the differential diagnosis of central and peripheral thrombocytopenia in dogs and cats
Source: J Vet Intern Med. 2024 Apr 15;38(3):1512–9. doi: 10.1111/jvim.17074 (PMC11099766; doi:10.1111/jvim.17074)
Supplement: Supplementary file 8 — Supplementary Table 5. Immature platelet fraction (IPF) in healthy cats grouped by age. [file JVIM-38-1512-s003.docx]

**Supplementary Table 5.** **Immature platelet fraction in healthy cats grouped by age.**

|  | **Healthy cats** | | |
| --- | --- | --- | --- |
|  | **Group 1:**  **<5 yo**  **(n=32)** | **Group 2:**  **5-10 yo**  **(n=22)** | **Group 3:**  **>10 yo**  **(n=12)** |
| IPF (%) | 17.3 ± 10 | 17.2 ± 9.7 | 12.4 ± 8.9 |
| IPFc (10^3^/µL) | 66.9 ± 42 | 65.7 ± 35 | 43.4 ± 28 |

Data are expressed as mean and SD. IPF, immature platelet fraction; IPFc, immature platelet count. ^*^P < .05 vs other groups.
