# Supplementary material for: Utility of immature platelet fraction in the Sysmex XN‐1000V for the differential diagnosis of central and peripheral thrombocytopenia in dogs and cats
Source: J Vet Intern Med. 2024 Apr 15;38(3):1512–9. doi: 10.1111/jvim.17074 (PMC11099766; doi:10.1111/jvim.17074)
Supplement: Supplementary file 9 — Supplementary Table 6. Correlations between platelet parameters in healthy (A) and thrombocytopenic (B) cats. [file JVIM-38-1512-s002.docx]

**Supplementary Table 4.** **Correlations between platelet parameters in healthy (A) and thrombocytopenic (B) cats.**

A)

|  | PLT (10^3^/µL) | IPF (%) | IPFc (10^3^/µL) |
| --- | --- | --- | --- |
| PLT (10^3^/µL) | 1.00 | - 0.11 | 0.35 |
| IPF (%) |  | 1.00 | 0.84 |
| IPFc (10^3^/µL) |  |  | 1.00 |

Data are expressed as Spearman’ or Pearson’ correlation coefficient, based on distribution. IPF, immature platelet fraction; IPFc, immature platelet count.

B)

|  | PLT (10^3^/µL) | IPF (%) | IPFc (10^3^/µL) |
| --- | --- | --- | --- |
| PLT (10^3^/µL) | 1.00 | 0.04 | 0.57 |
| IPF (%) |  | 1.00 | 0.77 |
| IPFc (10^3^/µL) |  |  | 1.00 |

Data are expressed as Spearman’ or Pearson’ correlation coefficient, based on distribution. IPF, immature platelet fraction; IPFc, immature platelet count.
