# Supplementary material for: Utility of immature platelet fraction in the Sysmex XN‐1000V for the differential diagnosis of central and peripheral thrombocytopenia in dogs and cats
Source: J Vet Intern Med. 2024 Apr 15;38(3):1512–9. doi: 10.1111/jvim.17074 (PMC11099766; doi:10.1111/jvim.17074)
Supplement: Supplementary file 10 — Supplementary Table 7. Cut‐off values of platelet parameters by receiver operating characteristic (ROC) curve analysis for the diagnosis of central thrombocytopenia in dogs and cats. [file JVIM-38-1512-s008.docx]

**Supplementary Table 7.** **Cut-off values of platelet parameters by** **receiver operating characteristic (ROC) curve analysis for the diagnosis of central thrombocytopenia in dogs and cats.**

|  |  | **Cut-off** | **Sensitivity**  **(%)** | **Specificity**  **(%)** | **AUC**  **(95% CI)** | **P value** |
| --- | --- | --- | --- | --- | --- | --- |
| **IPF** | *Dogs* | 6.90 | 95.1 | 94.6 | 0.982  (0.973 – 0 .992) | <0.0001 |
|  | *Cats* | 13.6 | 94 | 85.3 | 0.951  (0.908 – 0.993) | <0.0001 |
| **P-LCR** | *Dogs* | 34.0 | 69.1 | 43.4 | 0.5698  (0.519 – 0.620) | 0.0084 |

AUC, area under the curve; CI, confidence interval; IPF, immature platelet fraction; P-LCR, platelet-large cell ratio.
